# Vulnerability in procedural ethics: A study of 44 national research ethics guidelines

**DOI:** 10.1177/14687941251377266

**Published:** 2025-09-29

**Authors:** Rebecca Tapscott, Sophie Moxon

**Affiliations:** 1 3526 University of Glasgow, UK; 2 Independent Researcher, UK

**Keywords:** procedural ethics, vulnerability, qualitative research, research ethics review, informed consent

## Abstract

How should qualitative social scientists understand and engage with the concept of vulnerability in their research? Scholars have largely approached this question from the perspective of *ethics in practice*, inductively exploring the real-world challenges that arise when studying marginalised and subaltern communities. This article examines vulnerability from the perspective of *procedural ethics* – or the formal rules and processes that guide the regulation of research ethics – by examining the concept in 44 national research ethics guidelines from around the world. The findings show that, in contrast to the relational, embedded and dynamic understanding of vulnerability elaborated by qualitative social science scholars, national research ethics documents remain heavily dominated by understandings of vulnerability originating from biomedical research, both in terms of how they define and action vulnerability and the recommendations they propose to mitigate it. For guidelines that extend their remit to the social sciences, they increasingly include caveats and nuances that help speak to the concerns of qualitative social scientists. However, they do not question key underlying assumptions that continue to create challenges for qualitative social science researchers, especially those working in a critical tradition.

## Introduction

Research ethics regulations have been the subject of wide-ranging and longstanding criticisms, especially by qualitative social scientists, and particularly among ethnographers and field researchers. These regulations, sometimes called *procedural ethics* to distinguish them from the real-world ethical quandaries that researchers encounter,^1^ are widely recognised as emerging from and primarily suited to biomedical and clinical research (Gunsalus, 2002; Schrag, 2010; Whitfield, 2019). They are saturated with assumptions about what research is and what it is for that often clash with the reflexive nature of qualitative social science work.^2^ Although social scientists widely recognise this problem, and indeed, have relentlessly documented wide-ranging experiences of it, we still have surprisingly little systematic evidence to substantiate these criticisms and ward off those who suggest such complaints reflect individual bad experiences rather than system-wide issues (Bell and Wynn, 2023). Moreover, a systematic approach is helpful to understand the status quo of ethics regulations and to propose reform.

This article therefore conducts a systematic review of the concept of ‘vulnerability’ in ethics guidelines from 44 countries to consider if and how they speak to the concerns of qualitative social scientists. We use the term *qualitative social science* to encompass research that is concerned with social rather than biological questions, that uses qualitative methods, and that is defined by a degree of reflexivity. By reflexivity, we mean that social science researchers are embedded in and shape the social worlds they study, rather than autonomous from the natural world as in biomedical research. Relatedly, the topics that qualitative social sciences research must potentially be subject to critique about their social status and value. For example, studies of social phenomena such as migration, elections or gender equity must be able to reflect on whether these constitute social ‘goods’ or ‘bads’, for whom, and under what conditions; in contrast, studies of disease typically start from an assumed common goal of eradication (Tapscott, 2025; Whitfield, 2019). Qualitative social science is by no means a perfect term, but through it we seek to refer to some of the diverse disciplines, methods and topics of research that are so uncomfortably governed by existing dominant approaches to research ethics.

Vulnerability has increasingly been recognised as an important consideration in the conduct of qualitative social science research, for example, in studies that use ethnographic and field-based methods (see, e.g. Majic and Ditmore, 2024; Rodríguez and Minatti, 2024; Veloso et al., 2025 in this special issue). It is also an area that is insufficiently served by existing guidelines, for example, because they tend to see vulnerability as binary and static (Luna, 2015); fail to consider that vulnerability can be mutually felt and co-constituted by research participant and researcher (Bashir, 2020); and close off the epistemic value of such encounters (Minatti and Gass-Quintero, 2025).

Rather than present our own definition of vulnerability, this article highlights a discrepancy between the definitions adopted by qualitative social scientists and the national-level ethics guidelines that inform ethics review. Qualitative researchers may dismiss procedural ethics as bureaucratic red tape and a box checking exercise (Hammersley, 2009; Librett and Perrone, 2010; Wynn, 2011). However, such requirements increasingly govern research practice: nearly 90% of countries worldwide have adopted such regulations, setting standards for institutional-level ethics review processes that govern qualitative social sciences along with other forms of so-called human subjects research (Tapscott and Rincón Machón, 2024). These requirements shape research in its design, implementation and presentation. It is therefore important for qualitative social scientists to understand how these guidelines approach the concept of vulnerability. Such an understanding can help qualitative researchers navigate procedural ethics requirements and advocate for their reform.

Our findings show that national research ethics guidelines approach vulnerability through an epistemological orientation that understands the purpose of research as pursuing an uncontested good (human health and embodied welfare), and sees ethical research as determined by following a particular procedure (prior review by committee). Whether made explicit or left implicit, vulnerability is conceptualised as a lack of autonomy, or the inability of research participants to make free and informed decisions about whether to participate in research. Guidelines also propose ways to mitigate risks for vulnerable peoples. These are primarily (1) identifying a representative to act on behalf of the respondent, who can give free and informed consent on their behalf, whether that is assent in the case of minors, or third-party permissions, or (2) avoiding research on vulnerable populations, when possible. As with definitions of vulnerability, these solutions understand research participants as biologically embodied entities – such that it is feasible for a third-party to assess the interests of another and one's social identity is not core to the research question.

Guidelines that explicitly extend their scope to social sciences do not address these underlying foundational issues highlighted above, but rather speak to the problems that emerge as a result. These include, for example, broadening definitions of vulnerability in various ways, for instance, to encompass social factors such as educational or economic disadvantage; to consider the wider constellation of actors engaged in research, such as the researcher and research team; and to offer some flexibility in the collection of written informed consent. A selected few guidelines, such as Canada's and South Africa's, speak to the broader politics of knowledge production in ways that highlight the social and political construction of vulnerability. While helpful, these modifications maintain biomedical assumptions about what research is and what it is for, and the role of vulnerability therein, that remain limiting for some types of qualitative social science.

This article proceeds in four parts: First, it contextualises national-level ethics regulations and research ethics committees as implementing bodies and provides an overview of the disjunct between procedural ethics requirements and the concerns of qualitative social scientists when it comes to vulnerability. Second, it discusses the research methods. Third, it presents findings, exploring how national-level ethics guidelines define vulnerability and whether these definitions engage with questions of relationality, temporality and research context; whether they consider the broader constellation of actors involved in research, or focus solely on research participants; and whether their mitigation approaches reflect the distinct needs of the social sciences. Across these areas, the article further considers whether guidelines that focus on the social sciences have made greater accommodation than those nominally only concerned with biomedical and clinical research, and the extent to which this addresses the foundational issue highlighted above. The final section concludes with a reflection on implications for qualitative social sciences.

## Vulnerability in procedural and practical ethics

### Vulnerability in procedural ethics: international and national ethics guidelines

It has been widely recognised that there is a significant gap between how vulnerability is conceptualised in so-called ‘procedural ethics’ – or the formal rules on how to conduct ‘ethical’ research – and ‘ethics in practice’ – or the real-world ethical challenges that researchers encounter (Guillemin and Gillam, 2004). This is in no small part because international and national guidelines on research ethics emerge from and are designed for biomedical and clinical research. Our current blueprint for research ethics guidelines emerged in the aftermath of WWII to address concerns about medical and psychological experimentation on human bodies. Many of these stemmed from innovations in experimental medical research which required larger groups of participants to develop and test new drugs and treatments. Their concern with vulnerability is grounded in the once common practice of conducting experimental medical research on populations that could not refuse – whether due to poverty, mental or physical disability, imprisonment or institutionalisation. Origin stories often begin with Nazi experiments committed on prisoners during World War II, and continue through to studies of Black Americans and mentally disabled children in the United States in the 1960s and 1970s (see Heimer and Petty, 2010: 602–605 for a discussion on ‘IRB history: whig and revisionist’). A paradigmatic change in clinical research in the latter half of the 20th century, including the new practice of testing drugs on control populations in addition to clinically-ill patients, further fuelled concerns about the exploitation of vulnerable populations (Stark, 2011) including prisoners (Israel, 2016), and residents of lower-income countries who lack access to high quality healthcare (Silva et al., 2016; Viergever and Li, 2015).

Since their introduction, ethics regulations have been increasingly extended across all forms of scholarly research, including qualitative social sciences, and despite widespread objections from qualitative social scientists (Schrag, 2011; Tapscott, 2024; Tapscott and Rincón Machón, 2024). The content of ethics regulations, whether tailored to the social sciences or not, is therefore of concern to social scientists. However, existing research on ethics regulations are disproportionately from a biomedical standpoint (see, e.g. Bracken-Roche et al., 2017; Childress and Thomas, 2018; Eckenwiler et al., 2008; Gennet et al., 2015; Iltis et al., 2009). This brings with it implicit understandings of research as deductive, positivist and often – but not always – quantitative. As a result, studies often adopt a technocratic valence, assessing the suitability of said documents to uphold presumed universal and attainable ethical standards. However, scholars have long noted that the social sciences can raise distinctive ethical questions, having lower or at least different types of risks (Gunsalus, 2002); occurring in real-world and uncontrolled settings (Whitfield, 2019); concerned with group and social behaviour (Johnson, 2018; Whitfield, 2019); and tackling a wide variety of social questions for which there is no common guiding moral good or ultimate goal for research (Tapscott, 2025; Whitfield, 2019). It is therefore essential to analyse procedural ethics from the viewpoint of qualitative social scientists as a first step toward better understanding if and how they stand to shape qualitative social sciences, how we might more successfully navigate them, and reform them for the better.

### Vulnerability in practice: insights from qualitative social scientists

There is a rich and growing literature among qualitative social scientists on defining and navigating vulnerability in research practice, and relatedly, pointing to the shortcomings of procedural ethics in these endeavours. Key insights emphasise the need to move beyond a categorical approach to adopt definitions that recognise vulnerability as dynamic, relational and temporal; grounded in political and social contexts that can themselves change; and as relevant for a broad constellation of actors engaged in the research ecosystem rather than of relevance only to the research ‘subject’. Qualitative social scientists have also reflected on the need to challenge the ‘solutions’ offered by procedural ethics, including relying on signed informed consent and limiting research on vulnerable peoples.

An early insight from qualitative social scientists highlights a problematic tendency of procedural ethics approaches to use categories to determine whether or not a research participant is vulnerable. This ‘categorical approach’ relies on socially-formed assumptions about what makes an individual or group vulnerable, and presents vulnerability as static and decontextualised. As a result, it can be patronising and reinforce stigma against marginal groups, create barriers to participation in research, hinder research progress, and fail to adequately protect participants (Bracken-Roche et al., 2016; Shepperd, 2022; von Benzon and van Blerk, 2017). For example, von Benzon describes how parents and schools denied her access to children with learning disabilities, creating an obstacle for research designed to benefit these children; denying them an opportunity to contribute to a research agenda fundamentally about them; and limiting their ability to shape how others perceive them, thereby reinforcing existing stigmas (von Benzon and van Blerk, 2017). Categorical approaches to vulnerability can thus be a disservice to both researchers and participants (Grady, 2009; Luna, 2015; Rodríguez and Minatti, 2024). Instead, scholars variously propose understandings of vulnerability that account for the dynamic interplay between embodied vulnerabilities and socially-produced vulnerabilities, revealing vulnerability as complex and continually (re)negotiated. In a related critique, scholars have noted that vulnerability is not binary, but rather complex and multifaceted. Luna (2015), for example, proposes using the concept of ‘layered’ vulnerabilities, to better understand how diverse vulnerabilities can interact and compound.

In this sense, qualitative social scientists understand vulnerability as not a static category, but as relational, evolving and embedded in and shaped by a complex social environment. This means that the qualitative social scientist is often enmeshed in complex relationships with respondents, at times lacking full or even partial control. In this issue, Minatti and Gass-Quintero (2025) reflect on how respondents and researchers can mutually explore vulnerability in their relationship, as they exchange personal and emotional experiences – and how this shapes research findings, at times allowing richer and more nuanced understanding. Moreover, the nature and degree of vulnerability can evolve and change – whether due to changes in social or political environments (for instance, changes in government, as documented by Knott, 2019). Others explore how the research environment shapes vulnerability: for example, Malejacq and Mukhopadhyay discuss research in violent and dangerous settings, noting that field researchers can often only secure safe passage by building social community – or ‘tribes’ – with powerful and at times hostile actors, effectively inverting the biomedical assumption that research participants are vulnerable to researchers (Malejacq and Mukhopadhyay, 2016).

Qualitative social scientists have further highlighted the need to consider vulnerabilities beyond the research participant, considering those involved in the research ecosystem (e.g. interpreters, research brokers, research assistants); how they can become acutely vulnerable during the research process; and exploring dynamics within the research team itself (Baaz and Utas, 2019; Rodríguez and Minatti, 2024). Other scholars highlight how so-called ‘local’ research teams often bear the burden of physical and emotional risks of difficult and dangerous research, for example, conducting interviews in conflict zones (Ansoms et al., 2021; Cronin-Furman and Lake, 2018).

In terms of mitigating vulnerability, qualitative social scientists have cast doubt on the common requirement for signed informed consent. Wynn and Israel (2018) highlight three particular concerns with written informed consent in qualitative research: (1) the practice of securing informed consent once before research starts does not sit well with qualitative studies that evolve over time, and in which social relations are continually renegotiated and shaped by complex power dynamics; (2) it can be off-putting or even offensive to respondents. For example, in post-colonial contexts, signing a document was seen as coercive and associated with signing away rights rather than securing them; and (3) signed informed consent can be dangerous for participants, especially in politically charged or authoritarian settings, where it can constitute evidence of participation in research and threaten the promise of anonymity. Others note that signed informed consent can be weaponised to preclude critical research, for example on powerholders or elites (Storeng and Palmer, 2019).

A second common ‘solution’ to mitigate ethical problems of researching vulnerable people is to avoid it altogether. While it is undeniably important for qualitative social scientists to reflect on whether and how to engage vulnerable people directly in their research, the assessment often rests on a different set of considerations than in biomedical studies. As highlighted earlier, medical studies have a long history of experimenting on vulnerable people because they were easier to access and allowed researchers to justify a lower standard of care, thereby facilitating swift and low-cost research that could benefit the broader population. In such studies, researchers could assess risk to vulnerable participants as minor or consistent with the risks that participants would encounter in everyday life. For instance, in the Willowbrook hepatitis study, researchers infected mentally disabled children with hepatitis, arguing that it was endemic to the facility they were housed in, and the children would inevitably contract it anyway (it was, and they probably would have). The researchers justified the study as observation of a phenomenon that would occur anyway, even while the justificatory conditions of vulnerability were due to social rather than biological conditions (Rothman, 1982). In biomedical research, there is thus a clear rationale behind both avoiding the use of vulnerable populations and ensuring that they benefit from research. As Alex John London writes, the Belmont Report – which itself has influenced many ethical guidelines worldwide – indicates:

…that social justice requires that vulnerable groups not be chosen for inclusion in research simply because of their ‘easy availability, their compromised position, or their manipulability, rather than for reasons directly related to the problem being studied’ (National Commission for the Protection of Human Subjects of Biomedical and Behavioral Research, 1979) (London, 2022: 55).

However, qualitative research is often designed with the express purpose of understanding the perspectives of research participants and providing interpretive insights that rely on consideration of the individual in their context. Even more so, critical scholarship often intentionally selects marginalised or vulnerable populations as its subject in order to question hegemonic narratives. For example, feminist International Relations has advanced important arguments about how state authority and power are discursively and socially produced, by studying the otherwise hidden role that women play in its co-production (Enloe, 2014).

Such wide-ranging criticisms from qualitative social scientists about ‘vulnerability’ in procedural ethics are thus symptoms of broader foundational divergences between the epistemic assumptions underpinning ethics regulations and those of qualitative social science; and understandings of the purpose of research as pursuing a common end (embodied welfare) versus an open-ended, reflexive and critical exploration of the social. As Bell and Wynn highlight, ethics regulations conceptualise risk fundamentally differently from qualitative social scientists – these guidelines are premised on the idea that researchers’ interests are opposed to that of research participants; the very need for ethics regulation is premised on the idea that research participants need protection from researchers. In pursuit of this aim, they require researchers to speak a particular language of risks and benefits that do not correspond to ethnographic and field-based research, such that ethnographers cannot accurately represent their plans in ethics applications, and therefore rarely receive relevant feedback (Bell and Wynn, 2023). In a related but distinct critique, others have pointed out that ethics regulations rely on open-ended principles that are given meaning in relation to an underlying assumption or understanding of the purpose of the research – this is needed to determine, for example, what constitutes a harm or benefit, and relatedly, the stakes of being ‘vulnerable’ (Tapscott, 2025; Whitfield, 2019). But while biomedical and clinical research have a shared conception of research as promoting human health and longevity, there is no similar shared idea of the purpose of social scientific research. Instead, the objects of our enquiry are necessarily subject to normative contestation (Tapscott, 2025; Whitfield, 2019).

While there are evident gaps between how vulnerability is conceptualised and mitigated in procedural ethics versus ethics in practice, these findings are based on single cases and researchers’ personal experiences. They leave unanswered the question as to the breadth and depth of the problems identified, unable to speak to global trends, and subject to critiques that they are idiosyncratic or cherry pick problems from across heterogeneous systems (Hedgecoe, 2012). In 2007, Charles Bosk referred to this as a ‘chorus of complaint’ (Bosk, 2007); several years later, Zachary Schrag reflected that while this chorus is ‘louder than ever’ it is an ‘overall clamor’, remaining anecdotal and disciplinarily siloed such that no clear verse can be heard (Schrag, 2011: 121). Over a decade later, as the same ethics regulations are increasingly extended to the social sciences, the need for systematic evidence and interventions to better support and facilitate qualitative social sciences is apparent.

## Methodology

To fill this gap, this article presents a comparative and systematic study of 44 national ethics guidelines. We use a mixed-methods approach, combining quantitative analysis with a close reading of guidelines, because it helps to show the systemic nature of the disjunct between how these guidelines approach ethics and the concerns of qualitative social scientists. To determine the sample, the second author, Sophie Moxon,did a systematic Internet search for national research ethics guidelines available in English, using the search term: ‘[Country] national research ethics guidelines’. For each country, she then shortlisted one document that best met the following criteria: (1) The document was the most cited national guideline; (2) the document had the most universal application to different disciplines; (3) it was the most recent available version. As previously noted, national-level guidelines are generally oriented toward biomedical and clinical research, often remaining silent on social sciences (Tapscott and Rincón Machón, 2024). Absent a document tailored to the social sciences, biomedical guidance often becomes an important point of reference for researchers and regulators, including those working in the social sciences. We therefore selected the most salient document for social science researchers in each country; in some cases, this included documents that are nominally purely biomedical in scope.

The resultant sample includes 44 documents, representing Africa (13 countries), Asia (10 countries), the Caribbean and Oceania (3 countries), Europe (10 countries), the Middle East and North Africa (MENA (6 countries), and North America (2 countries), and with varying scopes: guidelines apply to biomedical and clinical research (10 countries); health-related research (13 countries); to the social sciences explicitly (13 countries); or to all research, and thus to the social sciences implicitly (8 countries) (see Table 1).

**Table 1.** Country guidelines according to their scope.

Biomedical and clinical research only *[10 countries]*		Health-related research (health-related research including social sciences) *[13 countries]*		Social science, implicit (all research with ‘human subjects’) *[8 countries]*		Social science, explicit (explicit reference to social sciences) *[13 countries]*	
Bahrain Botswana Kenya Liberia Oman	Saudi Arabia Sierra Leone Singapore Switzerland Turkey	Bangladesh Denmark Ethiopia Iceland India Japan Malta	Nepal Nigeria Tanzania UK Zambia Zimbabwe	Australia Cambodia Estonia Jamaica Malaysia Taiwan	UAE USA	Austria Canada Finland Lebanon Malawi	New Zealand Norway Philippines South Africa Sudan Sweden Thailand Uganda
							
							
							
							
							
							

The documents were also issued by different organisations, with 43% (19/44 documents) issued by research councils; 36% (16/44 documents) issued by government departments; and the remainder issued by other organisations including national science commissions or national health authorities. In our sample, documents pertaining only to biomedical and clinical research are more often issued by government departments or regulatory agencies (70%, or 7/10 documents), while those that extend to the social sciences implicitly or explicitly are more often issued by research councils (57%, or 12/21 documents).

### Content analysis

The study employs a content analysis, examining if and how selected documents use the term ‘vulnerability’ and what mitigation strategies they recommend. The first stage of data collection deductively coded a set of variables related to how the documents use the term vulnerability (e.g. whether the document explicitly defines vulnerability, whether examples of vulnerable groups or peoples are given, if it references the researcher or research team in relation to vulnerability). We also identified guideline-level characteristics (i.e. issuer, legal status and scope), and country-level characteristics (such as region). After finalising the coding framework, we manually coded each document,^3^ and conducted descriptive analysis in Stata. Throughout, we provide illustrative examples from selected documents.

### Limitations

While the research was designed to ensure robustness and reliability, it is important to highlight some key limitations. First, we include only English language documents. This mitigates concerns over how to interpret translations by third-party software, for example, Google Translate, but also limited our sample to countries with anglophone research traditions. Second, our sample includes only one national-level guideline per country. An analysis of additional guidelines could offer a thicker and more nuanced picture of what we set out below. Additionally, our findings reflect regulatory guidance, but do not necessarily speak to how ethical review is implemented in practice. Nonetheless, regulatory guidance sets standards in any given country and orients the practices of research ethics committees. It is thus an important area of study.

## Findings: ‘vulnerability’ in 44 national ethics guidelines

### Defining vulnerability in national-level guidelines

Although vulnerability is a key consideration in assessing the ethical stakes of research, fewer than half (43%, or 19/44) of countries offer an explicit definition of the term in their national guidelines. The United States – the progenitor of ethical review worldwide (Stark, 2011) – provides no explicit definition of vulnerability, nor do other research-leading countries, including Australia and the UK. Among guidelines that provide explicit definitions, they correspond closely to two international guidelines developed for medical research: the Council for International Organizations of Medical Sciences (CIOMS)'s International Ethical Guidelines for Biomedical Research Involving Human Subjects^4^ and the glossary of the International Council for Harmonisation's Good Clinical Practice (ICH-GCP).^5^ Both definitions refer specifically to a biomedical context and emphasise the ability of a research participant to give free and informed consent in relation to participation in a clinical trial (ICH-GCP) or lack of access to medical care (CIOMS).

Given that our sample includes guidelines from around the world, and a full half of them do not define vulnerability, the extent of similarity in definitions is striking. All but two documents in our sample emphasise capacity to give informed consent and/or freedom from undue influence in their definitions of vulnerability (the exceptions are South Africa and Malta). For example, Ethiopia's National Research Ethics Review Guidelines defines vulnerability as ‘having reduced capacity to offer free and informed consent due to possible coercion, undue influence, or other diminished autonomy’ (2014: 54), while Iceland's guidelines specify that vulnerable individuals are those ‘who for some reason are not in a position to make an informed or free decision’ (2014: 5). Several countries adopt the ICH-GCP definition word for word (Botswana, Sierra Leone, Turkey and Zimbabwe). Embedded in these definitions are elements of individualism, and as we elaborate subsequently, a particular positivist and non-reflexive understanding of research that is often incompatible with the epistemologies of qualitative social scientists.

A handful of guidelines have broader definitions that recognise vulnerability as a product of a particular set of power dynamics and an increased susceptibility to harm, rather than merely related to the capacity to consent and freedom from undue influence – these are all countries that extend regulation either to health-related research (India, Malta, Nepal) or explicitly to the social sciences (Canada, Philippines, South Africa). For example, India's National Ethical Guidelines for Biomedical and Health Research Involving Human Participants specify that vulnerability is not an inherent characteristic, but can be created through ‘situational conditions’ such as economic or social disadvantages (e.g. facing humanitarian emergencies or language barriers) (2017: 10). The National Ethical Guidelines for Health Research in Nepal note that, ‘Vulnerabilities should be seen broadly in terms of individuals adaptive capacity, exposure, and sensitivity which their economic status, position as migrants, age, and gender among other factors…’ (2022: 4). In addition to highlighting capacity to consent, the Philippine's National Ethical Guidelines for Health and Health-Related Research notes that ‘It also refers to the increased likelihood of being wronged or of incurring additional harm’ (2017: iv). By asking researchers to account for social dynamics, these definitions create space to consider vulnerability as a characteristic that is socially produced, and which therefore researchers and respondents contribute to constituting.

Canada's Tri-Council Policy Statement similarly emphasises capacity to consent in its explicit definition of vulnerability, but also offers further discussion of vulnerability throughout the document that speak to the insights of qualitative social scientists. For example, the document notes that ‘Individuals may experience vulnerability to different degrees and at different times, depending on their circumstances’ (2022: 279). The Statement also includes chapters on indigenous populations (Chapter 9) and qualitative research (Chapter 10), which offer further nuances and caveats to the concept of vulnerability – for example, noting that qualitative studies:

[S]ometimes engage in research that questions social structures and activities that create, or result in, inequality and injustice. Studies may involve participants whose circumstances make them highly vulnerable in the context of research because of the social and/ or legal stigmatization that is associated with their activity or identity, and who may have little trust in the law, social agencies or institutional authorities. Regardless of the methodological approach, researchers who question social structures or deal with the disempowered may face pressures from authority figures. Research may also involve participants, such as business executives or government officials, who may be more powerful than the researchers. (p. 185)

This statement recognises vulnerability as a potentially changing and evolving characteristic importantly constituted through social and political structures. However, the guidelines also ground vulnerability in capacity to consent and to be free from undue coercion, and advise seeking consent from ‘an authorized third party’ who can speak to the respondent's wishes and/or welfare (p. 7). Here we therefore see an example of guidelines that have sought to address the insights of qualitative social scientists by extending their discussion of vulnerability, but without explicitly addressing foundational disjuncts, in which following ethics procedures may themselves create vulnerabilities, for instance cases where signing consent documents is what puts a respondent at risk (Bell and Wynn, 2023: 548). Perhaps unsurprisingly then, as we discuss subsequently in the section on informed consent, the guidelines apparently have not translated into widespread changes in practice by Canada's ethics committees, which instead continue to hew toward institutional risk-aversion by limiting research with vulnerable peoples and requiring signed prior informed consent (Bell, 2016; Bell and Wynn, 2023; Tauri, 2018).

Beyond the Canadian example, only two countries in our sample offer definitions that are substantially distinct from the CIOMS and ICH-GCP definitions: Malta and South Africa. Malta's Guidelines for Ethical Practice and Research Integrity define vulnerability as lacking social power (2022: 7), while South Africa's guidelines specify:

Vulnerability is not an absolute condition but rather occurs on a sliding scale. In South Africa, arguably, the majority of potential research participants are vulnerable when compared to those in North America or Europe, from whence much funding is sourced. Whether vulnerability is present is a matter of fact and degree. (2015: 26)

This definition both pushes back against a categorical approach to vulnerability (see the next section), and points to the political economy of research, contrasting who chooses research priorities with who supplies the ‘subjects’ needed to realise them. From this perspective, the definition highlights that South African research participants are in most cases not the primary beneficiary of research, as it is conceptualised and funded by actors based in the Global North. Thus, while the definition puts social sources of vulnerability front and centre, and even raises questions about the political economy of knowledge production, it still does not explore vulnerability as social constructed, instead retaining a biomedical emphasis – that South Africans are necessarily more vulnerable than North Americans or Europeans, an assumption that makes less sense for studies concerned with South Africans not as biological entities but as socio-political actors.

This analysis shows the heavy influence of biomedical understandings of vulnerability, with nearly all reviewed documents understanding vulnerability as a lack of autonomy. Those few countries that have extended their definitions of vulnerability point toward some of the insights of qualitative social scientists, but have yet to highlight the multiple angles in which vulnerability is complex, relational and dynamic.

### Vulnerable groups

**Figure 1.**
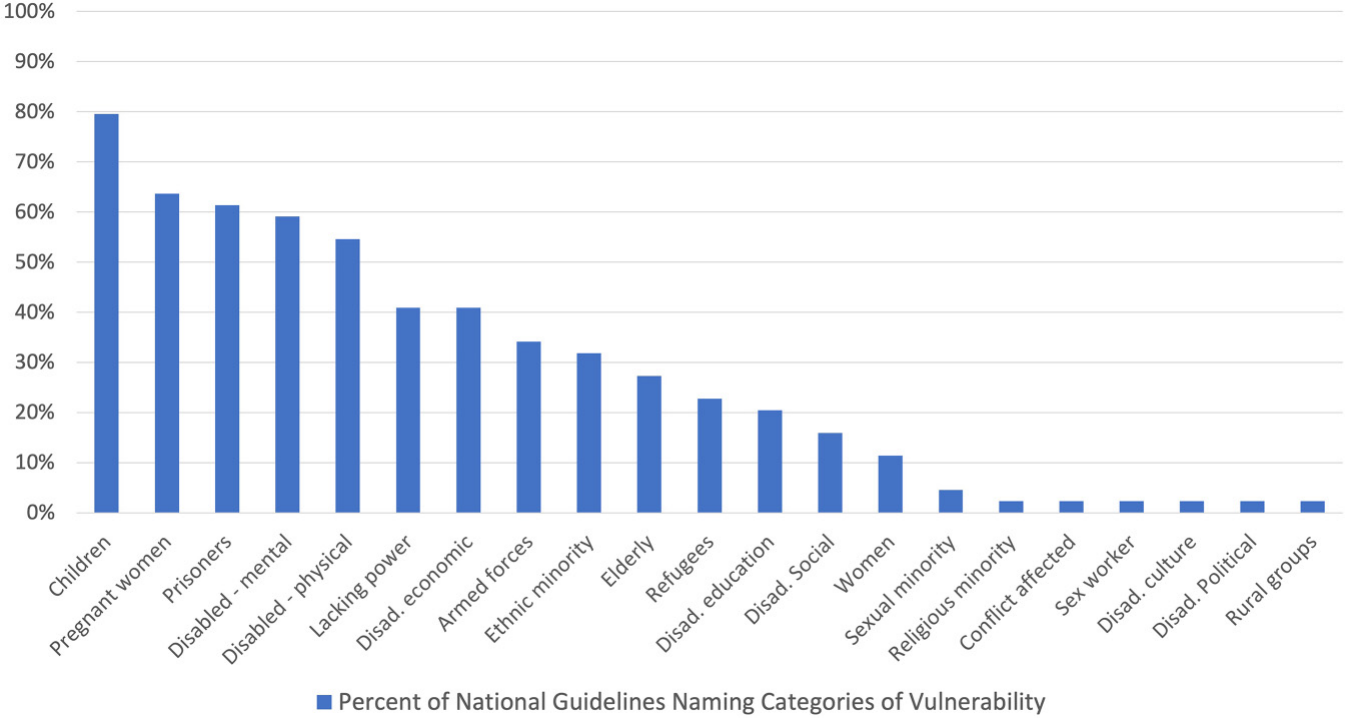
References to vulnerable groups across national guidelines.

More often than defining vulnerability, guidelines give examples of vulnerable groups or list conditions that may make people vulnerable. We found that 82% of guidelines (36/44) offer examples of vulnerable groups.^6^ Of these, 59% provide (non-exhaustive) examples of specific groups, for example, children or mentally disabled people, while 23% mention general circumstances that could cause vulnerability, for example, low literacy or poor access to healthcare. The most cited vulnerable groups are children (80% of guidelines), pregnant women/foetuses (64%), prisoners (61%), mentally disabled people (59%), and physically disabled people (55%) (Figure 1). Overall, we found 21 group types across our sample. Only 2% of guidelines give examples of non-institutionalised groups that are vulnerable primarily because of social or political marginalisation, for example, conflict-affected persons, sex workers and culturally or politically disadvantaged groups (Figure 1).

As illustrated in Figure 1, many guidelines include categories based on both embodied and social vulnerabilities. For example, the commonly listed categories of ‘prisoners’ and ‘armed forces’ highlight the social production of vulnerability, and how this has been central to the conduct of medical experimentation and advancement throughout the 20th and 21st century. While recognising social factors as important, the vast majority of guidelines do little to address how diverse vulnerabilities may interact, evolve and manifest differently depending on the environment as well as nature of the research. Instead, they translate diverse types of vulnerabilities into categorical examples that can be applied in a regulatory sense, but which suffer from a variety of critiques. There are some notable exceptions. For example, Kenya's National Guidelines for Ethical Conduct of Biomedical Research Involving Human Participants states:

In many developing countries including Kenya, prison conditions are very harsh, i.e., low quality or inadequate diet, poor linen and accommodation. The prisoners are always under fear of reprisals from the prison wardens if they do not comply with any instructions given to them. However, prisoners with serious illness or at risk of serious illness, e.g., HIV/AIDS, hepatitis, cancer and TB should not be denied access to investigational drugs, vaccines or other agents that show promise of therapeutic or preventive benefit. (2020: 17–18)

This text highlights both an obligation to limit use of prison populations, as prisoners may feel obliged to participate in a study if asked, but also an obligation not to exclude prisoners from studies as they may otherwise not be able to access therapeutic treatments. By underscoring the complex relationship among different types of vulnerabilities (institutional and embodied), this discussion emphasises why it is not advisable to adopt blanket rules for researching vulnerable groups.

### Vulnerability beyond the research ‘subject’

**Figure 2.**
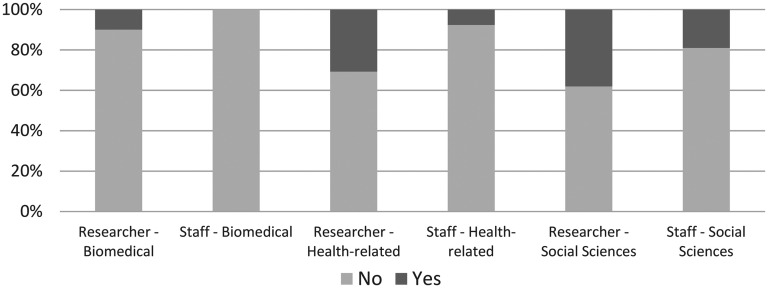
Do guidelines discuss vulnerability of broader research community? by document scope.

Across the board, guidelines focus on the vulnerability of the research participant, typically autonomously from the broader socio-political context. Most guidelines (70%) do not discuss the broader constellation of actors engaged in the research process, such as the researcher and their research team (e.g. enumerators, interpreters, lab techs, research assistants). Approximately 30% (13/44) of guidelines discuss the need to protect researchers, while only 11% (5/44) discuss the research team.^7^ Of guidelines that reference researcher vulnerability or risk, or that of the wider research team, most were from guidelines that implicitly or explicitly apply to the social sciences (Figure 2).

Most guidelines focus exclusively on vulnerability of the research ‘subject’. In this sense, guidelines’ broad silence on the constellation of actors that engage in the research process highlights underlying assumptions of a biomedical research model, which assumes the researcher can be autonomous from the research process.

Among guidelines that discuss risk to researchers, there was significant variation in the extent of consideration given to this issue. For instance, some guidelines (e.g. Austria and Estonia) made singular references to protecting researchers in a human resources context, ensuring researchers have access to a safe working environment. Other guidelines (e.g. Canada, Norway and India) include robust discussions of several associated ethical issues, such as the vulnerabilities of student researchers, different forms of harm that could befall researchers (e.g. physical and mental), the responsibilities of ethics committees and institutions to protect staff, specific circumstances that could exacerbate researcher vulnerabilities (e.g. emergency or conflict situations), and uncomfortable power dynamics between researchers, local researchers, interpreters and elite participants. For instance, Norway's Guidelines for Research Ethics in the Social Sciences and the Humanities (2021), which focus exclusively on the social sciences, discuss vulnerability of researchers as well as the wider research team:

Research may involve high risk, not just to the researchers but also to students, collaborators, research participants, co-workers, and interpreters. They may be endangered due to their participation in research beyond direct physical and mental harm by facing threats to their safety and well-being…Researchers are responsible for assessing their own safety and for not exposing partners and participants to unacceptable risks. (2021: 16–17)

While a selected few documents have begun to look beyond risk for those who participate in research, they majorly do so by extending their requirements to assess risk for other actors engaged in the research ecosystem. While important, this is majorly about risk, rather than engaging with the idea of vulnerability in research as a complex, relational and emerging dynamic, nor does it provide space to consider how said relations may evolve (even in non-linear ways) over time. Rather, by continuing to employ the language or risk and its mitigation, these reforms take on an expansionist logic, further securitising the research environment and making it suitable to risk assessments and legal liability checks. Moreover, even among those guidelines that recognise vulnerability of researchers and staff, the very design of prior ethics review imposes important limitations by asking researchers to identify vulnerabilities at the outset of research.

## Mitigation strategies and safeguards for vulnerable people and populations

### Informed consent as a safeguard for vulnerable populations

To mitigate potential harms associated with vulnerabilities, national ethics guidelines typically turn to informed consent procedures. This reinforces the finding that guidelines (1) understand vulnerability as a question of autonomy; (2) that can be redressed through informed consent; and (3) that therefore is particularly relevant to the early stages of a study when ‘subjects’ are ‘recruited’ and ‘enrolled’. By understanding vulnerability as an issue linked to the decision to participate in research, these guidelines draw a temporal limit around the researcher's obligations to identify and mitigate vulnerability and point toward specific procedural solutions, linked to ensuring that participants are informed and/or their interests are reflected for example by a third party (e.g. a caretaker).

**Figure 3.**
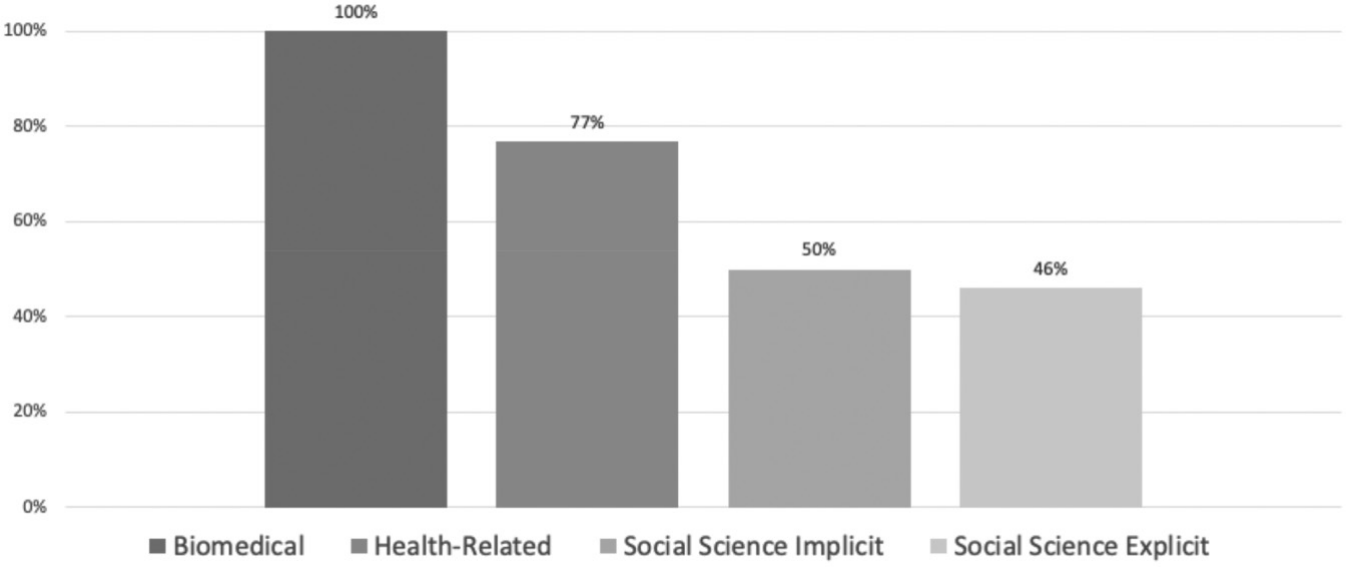
Requirement to document informed consent by document scope.

In addition to requiring informed consent, guidelines in our sample showed a heavy preference that this be documented, most typically through the use of signed consent forms; however, this differed depending on whether the documents were solely concerned with biomedical and clinical research or if their scope also extended to the social sciences. We found that while 100% of documents focused exclusively on biomedical studies and clinical trials require informed consent signed by research participants, this halved for documents that extend their mandate to the social sciences (Figure 3). Nonetheless, this still means that half of guidelines that extend their scope to the social sciences assume documented informed consent as standard practice. This is notable, given longstanding scholarship that highlights situations in which written informed consent can actually exacerbate vulnerabilities and harms rather than mitigating them.

Canada's guidelines provide the most discussion on how informed consent might be approached differently in different kinds of research, including a chapter dedicated to qualitative research (pp. 154–196) that discuss the timing and nature of consent, though not tailored to vulnerable populations specifically. The Statement provides for pilot research to be conducted prior to ethics approval so that researchers can inductively develop research questions (188–189), and different modalities of informed consent, including provisions for observational research (190–191). While speaking to the concerns of qualitative social scientists, they nonetheless continue to do so from the standpoint of a document still seeped in bioethical assumptions and concerned with auditability. For instance, while the document provides for elites speaking in their professional capacity to ‘signify consent to participate in the research’ it also notes that:

The researcher should record this in an appropriate way. Researchers shall demonstrate to the REB that the participant will be informed about the research and about the options to withdraw from the study at any time or not to participate at all. Nothing in this article should be interpreted to mean that prospective participants need not be informed about the study prior to their participation. (2022: 190)

Read in conjunction with the prior chapter on research with indigenous populations (pp. 146–182), Canada's guidelines address many criticisms raised by qualitative social scientists, while also illustrating some limitations of doing so in a way that does not contradict or undermine regulations for biomedical research. This appears to be borne out in practice, in that Canadian scholars and indigenous populations continue to lament how ethics committees implement guidelines conservatively, requiring documented informed consent as a default and disregarding the input of indigenous populations, despite these considered provisions (Tauri, 2018).

**Figure 4.**
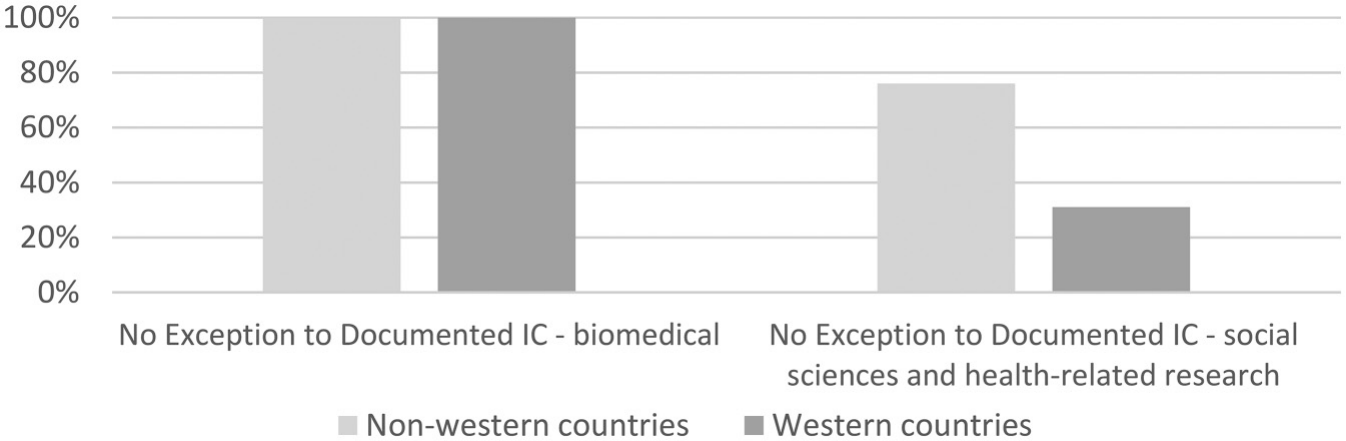
Requirement to document informed consent in ‘western’ versus ‘non-western’ countries.

Beyond a preference for written informed consent, we also find that countries outside of Europe, North America, Australia and New Zealand are *less likely* to offer exceptions to the collection of written informed consent (Figure 4).^8^ For documents that extend their scope beyond biomedical research, 76% (16/21) of non-western countries offer no exception to the collection of documented consent, compared to 31% of western countries (4/13). As highlighted above, all documents that limit their scope to biomedical studies insist on obtaining written informed consent.

This is especially concerning given that some of the key concerns with collecting documented informed consent relate to using this approach in non-western contexts. It also highlights that of those countries that adapt requirements for signed informed consent in national guidelines that apply to the social sciences, most of the adaptation is occurring in Europe and North America. In contrast, ‘non-western’ countries more frequently use the same requirements for consent across biomedical and social science research. This raises a concern for those who encourage the use of so-called ‘local’ ethics guidelines, with the hope that they more authentically reflect the interests and concerns of researched populations, in that they may reinforce other problematic aspects of procedural ethics when it comes to regulating qualitative social sciences.

### Obligation to include or exclude vulnerable groups

Some guidelines also ask researchers to mitigate potential harms to vulnerable populations by adopting research designs that either guarantee equal opportunity to participate, or proactively seek alternatives to vulnerable populations. Of the 19 countries that address this question, eight stipulate that vulnerable groups should only be included if alternatives are not possible, while four specify that vulnerable groups should have equal opportunities to participate in research. Seven countries require both the inclusion *and* exclusion of vulnerable groups (see Table 2).

**Table 2.** Obligation to include or exclude.

Obligation to include	Obligation to include and exclude	Obligation to exclude
Australia (Implicit social sciences)	Ethiopia (Health related)	Bangladesh (Health related)
Bahrain (Biomedical)	Oman (Biomedical)	Kenya (Biomedical)
Canada (Explicit social sciences)	Philippines (Explicit social science)	Nepal (Health related)
India (Health related)	Sierra Leone (Biomedical)	Nigeria (Health related)
	South Africa (Explicit social science)	Saudi Arabia (Biomedical)
	Sudan (Explicit social science)	Singapore (Biomedical)
	Tanzania (Health related)	Switzerland (Biomedical)
		Uganda (Explicit social science)

The tendency to mitigate vulnerability by limiting research on vulnerable groups is especially concerning for qualitative social sciences, as highlighted in the literature review. However, many guidelines oblige researchers only to research vulnerable populations if benefits will accrue to them. For example, Tanzania's Guidelines on Ethics for Health Research stipulate:

Vulnerable groups should not be targeted for high-risk research and should not be denied potentially beneficial research. (2023: 35)

Similarly, Ethiopia's guidelines stipulate an obligation to research and *not* to research vulnerable groups:

Scientific objectives, not vulnerability or privilege, and the potential for and distribution of risks and benefits, should determine communities selected as study sites and the inclusion criteria for individuals… Persons with disabilities (mental or physical) should not be unfairly excluded from participating in research. (2014: 16)

Vulnerable population involvement requires further explanation to justify that without these vulnerable populations involvement there is no other way of obtaining the relevant data. (2014: 47)

It is possible, if a bit awkward, to translate these guidelines to qualitative social sciences. At first blush, most social scientists will agree that so-called ‘vulnerable people’ should not be used as convenience samples, and that it is important to consider whether the research will meaningfully contribute new knowledge. However, when vulnerabilities are understood first and foremost as social in nature, problems begin to arise. First, most social scientists would argue that *no one* should be used as a convenience sample, both on ethical and methodological grounds, as the social rather than biological nature of our questions tend to mean populations and people are not interchangeable. This is especially true in contexts where power dynamics make research participants ‘vulnerable’ to the research or researcher. Moreover, determining whether a vulnerable person or population should be included or excluded on the grounds of their vulnerability requires a researcher to accept a given social construction of vulnerability as part of their study design. For example, if a researcher is studying a socially stigmatised group (say, children with learning disabilities), they can only establish whether excluding these children would be ‘unfair’ by accepting socially-constructed understandings of learning disabilities as a source of vulnerability and marginalisation. More broadly, this suggests that a critical perspective on the construction of vulnerability is usually, or should be, embedded in qualitative social science research from the outset.

## Conclusion

The analysis highlights that national-level ethics guidelines remain tethered to the assumptions of biomedical and clinical research. This is the case both for those that restrict their scope to biomedical and clinical studies, and to a large extent, those that extend it to the social sciences, whether implicitly or explicitly. This makes them a poor fit for qualitative social sciences, which are reflexive, and typically understand vulnerability as socially produced, relational and changing. Qualitative social scientists view themselves as embedded in the research field, co-producing vulnerability and resilience through the research process and outputs, as much as research participants. In research that seeks to understand society, the socially produced nature of vulnerability is more than a factor for researchers to consider, but is itself central to research. Consider critical scholarship, which frequently employs qualitative methods to build novel theory and explanation. This area often explicitly seeks to study the vulnerable to challenge hegemonic narratives, turning on its head the notion that it is ethical to protect ‘vulnerable populations’ *from* research (see, e.g. Das, 2006; Enloe, 2014; Tickner, 2011). In a different vein, research on powerful institutions and peoples arguably demands an understanding of *invulnerability*, its similarly relational, evolving and embedded characteristics, and different ethical commitments that might accompany that (Blee, 1993; Siddiqui and Turnbull, 2024).

In contrast, we found that guidelines remain firmly rooted in a biomedical tradition, even if they add nuances or caveats to speak to the issues that arise from this and that have been repeatedly highlighted by qualitative social scientists. The core definition of vulnerability that guidelines start with are the notions of autonomy and informed consent, specifically, whether participants have the capacity to consent to participate in research and whether they can be sufficiently informed. Building from this, most guidelines continue to conceptualise vulnerability as a static category rather than a dynamic characteristic that is shaped by (and shapes) an array of changing, contextual and layered factors. The most cited categories (children, pregnant women, prisoners and mentally disabled persons) are widely discussed as inherently vulnerable rather than as vulnerable in certain circumstances and due to socially produced and imposed contextual factors. This is a particularly poor fit for social scientists studying systems of power and oppression, or who wish to focus on marginalised and subaltern communities in order to bring their experiences to light. Moreover, it sits poorly with many qualitative methods, for example ethnographic and field-based methods, that require the researcher to engage with respondents and co-constitute complex and changing power dynamics. Additionally, national ethics guidelines rarely consider vulnerability beyond the research participant. Limited discussion is granted to the vulnerabilities that could befall the researcher, broader research team or researched communities as a result of research. This is particularly problematic for qualitative social scientists, whose very epistemologies and methods reflexively consider the role of the researcher, research team and even society in the political economy of knowledge production (Bell and Wynn, 2023; Tapscott, 2025).

The solutions that national guidelines offer to mitigate vulnerabilities also reflect their biomedical orientation, perhaps even more acutely. For example, the focus on securing documented informed consent limits concerns with vulnerability to the moment of obtaining consent, even while qualitative scholars have observed the changing dynamics of consent throughout the research process (Plankey-Videla, 2012). There is also a clear emphasis on auditable (documented and signed) consent processes, even while scholars have noted that obtaining written consent can be impractical and off-putting, introduce new harms, for example, related to security, and assumes a clear start and end to research that does not match qualitative methods such as ethnography (Wynn and Israel, 2018). It also precludes many critical studies of powerful actors and institutions, requiring their consent to make even critical investigations (Spicker, 2011). Emphasising inclusion and exclusion criteria as a response to vulnerability also has been extended, rather than rethought, for qualitative research. Only targeting vulnerable people in research if necessary, and at the same time ensuring their inclusion if it will offer direct benefits, is logically a key question to ask of biomedical and clinical research, which otherwise might exploit the vulnerable for the benefit of the privileged, as has occurred in numerous ethical controversies around the world. However, in well-designed qualitative research, such considerations should be central to the selection of research participants – as they often seek to better understand the perspective of a given group or engage them with the very purpose of troubling hierarchical and exclusive social orders.

Examining vulnerability as it appears in national-level ethics guidelines from around the world therefore highlights that procedural ethics remain designed for biomedical and clinical research, helping explain why guidance around vulnerability continues to translate poorly to many types of qualitative social science research, especially methods such as ethnography and fieldwork, and critical traditions. Many countries have added text and made select adjustments so they can plausibly apply to social sciences. Identifying and using these caveats and clarifications can help qualitative researchers better navigate procedural ethics requirements. However, our analysis shows that despite some modifications, the underlying blueprint and associated biomedical assumptions remain unchallenged.

While qualitative scholars should applaud and encourage adaptations and adjustments that take qualitative methods and epistemologies into account, meaningful change demands the development of ethics norms and procedures that build from the foundations of qualitative methods, rather than from bioethics (see, e.g. Bell and Wynn, 2023). This would require significant investment, time and resources; they are a long-term, not a short-term, solution. Qualitative researchers can also adopt more immediate strategies, for example, familiarising themselves with aspects of national guidelines that provide caveats and nuances sympathetic to our methods, epistemologies and disciplines; highlight and challenge the limitations of existing guidelines; and continue to advocate for change.

## Supplemental Material

sj-docx-1-qrj-10.1177_14687941251377266 - Supplemental material for Vulnerability in procedural ethics: A study of 44 national research ethics guidelines Supplemental material, sj-docx-1-qrj-10.1177_14687941251377266 for Vulnerability in procedural ethics: A study of 44 national research ethics guidelines by Rebecca Tapscott and Sophie Moxon in Qualitative Research

## Notes

1. For more on the distinction between ethics in practice and procedural ethics, see Guillemin and Gillam (2004).

2. For the epistemic point, see Bell and Wynn (2023); for the purpose of research, see Whitfield (2019) and Tapscott (2025); for a summary of critiques, see Schrag (2011).

3. These were checked by a second research assistant. We then compared these codes to test similarity, Statistical tests (Cohen's Kappa) were conducted to establish the level of agreement between the two coders. The statistical tests showed a high level of agreement between the coders across all variables, indicating that the data is reliable and there is minimal risk of human error in the dataset.

4. CIOMS, an international organisation established by the World Health Organisation (WHO) and the United Nations Educational, Scientific and Cultural Organization (UNESCO) in 1949 to advance public health, defines vulnerability as ‘a substantial incapacity to protect one's own interests owing to such impediments as lack of capability to give informed consent, lack of alternative means of obtaining medical care or other expensive necessities, or being a junior or subordinate member of a hierarchical group’ (2002: 11).

5. The ICH was established in 1990 to harmonise technical guidelines and requirements for pharmaceutical product registration, especially for clinical trial requirements. The ICH-GCP defines vulnerable subjects as: ‘[i]ndividuals whose willingness to volunteer in a clinical trial may be unduly influenced by the expectation, whether justified or not, of benefits associated with participation, or of a retaliatory response from senior members of a hierarchy in case of refusal to participate’ (2023: 56–57).

6. Countries that provide no examples of vulnerable groups are Austria, Cambodia, Estonia, Japan, Liberia, Malaysia, Sweden and the UK.

7. The following country guidelines discussed the need to protect researchers: Australia, Austria, Bangladesh, Canada, Estonia, India, Kenya, Malta, Nigeria, Norway, Philippines, South Africa and Uganda; Canada, Estonia, India, Norway and South Africa refer to vulnerabilities and/or risks to research staff.

8. In our analysis, we coded Australia, Austria, Canada, Denmark, Estonia, Finland, Iceland, Malta, New Zealand, Norway, Sweden, Switzerland, the United Kingdom and the United States as ‘western’ and Bahrain, Bangladesh, Botswana, Cambodia, Ethiopia, India, Jamaica, Japan, Kenya, Lebanon, Liberia, Malawi, Malaysia, Nepal, Nigeria, Oman, Philippines, Saudi Arabia, Sierra Leone, Singapore, South Africa, Sudan, Taiwan, Tanzania, Thailand, Turkey, Uganda, United Arab Emirates, Zambia and Zimbabwe as ‘non-western’.
