# Supplementary material for: Vulnerability in procedural ethics: A study of 44 national research ethics guidelines
Source: Qual Res. 2025 Sep 29;26(3):513–38. doi: 10.1177/14687941251377266 (PMC13245854; doi:10.1177/14687941251377266)
Supplement: sj-docx-1-qrj-10.1177_14687941251377266 - Supplemental material for Vulnerability in procedural ethics: A study of 44 national research ethics guidelines [file sj-docx-1-qrj-10.1177_14687941251377266.docx]

SUPPLEMENTARY MATERIALS

**A: National Research Ethics Guidelines**

**Australia**

National Health and Medical Research Council (2018) National Statement on Ethical Conduct in Human Research. [Online] <https://www.nhmrc.gov.au/about-us/publications/national-statement-ethical-conduct-human-research-2007-updated-2018> [Accessed 30 September 2023]

**Austria**

Austrian Federal Ministry of Education, Science and Research (2023) Best Practice Guide for Research Integrity and Ethics [Online] <https://www.bmbwf.gv.at/dam/jcr:7b02199c-96e2-446b-9f30-47e052d77c9c/> [Accessed 20 September 2023]

**Bahrain**

National Health Regulatory Authority (2016) Standards & Requirements for Independent Research Ethics Committee (IREC) Involved in Clinical Trials in the Kingdom of Bahrain. [Online] <https://nhra.bh/departments/cpd/ct/MediaHandler/GenericHandler/documents/departments/CT/Regulation/Annex%20II%20Standards%20and%20Requirements%20for%20IRECs.pdf> [30 September 2023]

**Bangladesh**

Bangladesh Medical Research Council (unknown) Ethical Guidelines for Conducting Research Studies Involving Human Subjects [Online]

<https://bmrcbd.org/application_form/EthicalGuidelines.pdf> [Accessed 30 September 2023]

**Botswana**

Ministry of Health and Wellness (2012) Guidelines for Regulating the Conduct of Clinical Trials Using Medicines in Human Participants. [Online]

<https://www.moh.gov.bw/Publications/drug_regulation/CLINICAL%20TRIAL%20GUIDELINES%20botswana%20v4-060312.pdf> [Accessed 30 September 2023]

**Cambodia**

National Council of Science, Technology & Innovation (2022) Research Ethics. [Online] [Accessed 30 September 2023].

**Canada**

Tri-Council (2022) Tri-Council Policy Statement: Ethical Conduct for Research Involving Humans – TCPS 2 [Online] <https://ethics.gc.ca/eng/policy-politique_tcps2-eptc2_2022.html> [Accessed 30 September 2023].

**Denmark**

The Danish Parliament (2018) Act on Research Ethics Review of Health Research Projects [Online] <https://www.retsinformation.dk/eli/lta/2011/593> [Accessed 30 September 2023]

**Estonia**

University of Tartu (2017) Estonian Code of Conduct for Research Integrity. [Online] <https://ut.ee/sites/default/files/inline-files/code_of_conduct_for_research_integrity_eng_1.pdf> [Accessed 30 September 2023]

**Ethiopia**

FDRE Ministry of Science and Technology (2014) National Research Ethics Review Guideline. [Online] <https://www.ccghr.ca/resources/harmonization/ethiopia/ethiopia-research-ethics/> [Accessed 30 September 2023].

**Finland**

National Advisory Board on Research Ethics (2009) Ethical principles of research in the humanities and social and behavioural sciences and proposals for ethical review. [Online] <https://tenk.fi/sites/tenk.fi/files/ethicalprinciples.pdf> [Accessed 30 September 2023].

**Iceland**

Ministry of Welfare (2014) Act on Scientific Research in the Health Sector. [Online] <https://www.government.is/media/velferdarraduneyti-media/media/acrobat-enskar_sidur/Health-Sector-Research-Act-No-44-2014.pdf> [Accessed 30 September 2023].

**India**

Indian Council of Medical Research (2017) National Ethical Guidelines for Biomedical and Health Research Involving Human Participants. [Online] <https://ethics.ncdirindia.org/asset/pdf/ICMR_National_Ethical_Guidelines.pdf> [Accessed 30 September 2023].

**Jamaica**

Ministry of Health and Wellness (2010) Guidelines for the Conduct of Research on Human Subjects. [Online] <https://www.moh.gov.jm/guidelines/guidelines-for-the-conduct-of-research-on-human-subjects/> [Accessed 30 September 2023].

**Japan**

Ministry of Education, Culture, Sports, Science and Technology (2015) Ethical Guidelines for Medical and Health Research Involving Human Subjects. [Online] <https://www.mhlw.go.jp/content/000757566.pdf> [Accessed 30 September 2023].

**Kenya**

National Commission for Science, Technology and Innovation (2020) National Guidelines for Ethical conduct of Biomedical Research Involving Human Participants in Kenya. [Online]

<https://www.nacosti.go.ke/nacosti/Docs/QUICK%20DOWNLOADS/National%20Guidelines%20for%20Ethical%20Conduct%20of%20Biomedical%20Research%20Involving%20Human%20Participants%20in%20Kenya.pdf> [Accessed 30 September 2023].

**Lebanon**

National Council for Scientific Research (2016) Charter of Ethics and Guidelines Principles of Scientific Research in Lebanon. [Online]

<http://www.cnrs.edu.lb/Library/Files/Uploaded%20Files/Charter_of_Ethics_En.pdf> [Accessed 30 September 2023].

**Liberia**

Liberia Medicines & Health Product Regulatory Authority (2020) Guidelines on the Conduct of Clinical Trials in Liberia [Online] <https://www.lmhra.gov.lr/new/pg_img/GUIDELINES%20ON%20THE%20CONDUCT%20OF%20CLINICAL%20TRIALS%20%2001.12.22.pdf> [Accessed 30 September 2023].

**Malawi**

National Commission for Science and Technology (2003) The Framework of Requirements and Guidelines for Research in the Social Sciences and Humanities in Malawi. [Online] <https://www.ncst.mw/wp-content/uploads/2014/03/NATIONAL-FRAMEWORK-OF-GUIDELINES-IN-SSH.pdf> [Accessed 30 September 2023].

**Malaysia**

National Science Council (2020) The Malaysian Code of Responsible Conduct in Research. [Online]

<https://www.akademisains.gov.my/asm-publication/the-malaysian-code-of-responsible-conduct-in-research-2nd-edition/> [Accessed 30 September 2023].

**Malta**

Malta Further and Higher Education Authority (2022) Guidelines for Ethical Practice and Research Integrity. [Online]

<https://mfhea.mt/wp-content/uploads/2022/08/Final-version_Ethics-Publication-Insert-Pages-Pages-1-12-Definition.pdf> [Accessed 30 September 2023].

**Nepal**

Nepal Health Research Council (2022) National Ethical Guidelines for Health Research in Nepal. [Online] <https://nhrc.gov.np/wp-content/uploads/2022/04/National-ethical-guidelines-4-Aug-28-2022.pdf> [Accessed 30 September 2023].

**New Zealand**

Health Research Council of New Zealand (2017) HRC Research Ethics Guidelines. [Online] <https://gateway.hrc.govt.nz/funding/downloads/HRC_research_ethics_guidelines.pdf> [Accessed 30 September 2023].

**Nigeria**

National Health Research Ethics Committee of Nigeria (2007) National Code of Health Research Ethics. [Online] <http://www.nhrec.net/nhrec/NCHRE_Aug%2007.pdf> [Accessed 30 September 2023].

**Norway**

The Norwegian National Research Ethics Committees (2022) Guidelines for Research Ethics in the Social Sciences and the Humanities. [Online]

<https://www.forskningsetikk.no/en/guidelines/social-sciences-and-humanities/guidelines-for-research-ethics-in-the-social-sciences-and-the-humanities/> [Accessed 30 September 2023].

**Oman**

Centre of Studies and Research (2016) Guidelines for Responsible Conduct of Clinical Studies and Trials. [Online]

<https://mohcsr.gov.om/wp-content/uploads/2016/01/Guide_ClinicalStudiesTrials_Aug16.pdf> [Accessed 30 September 2023].

**Philippines**

Philippine Health Research Ethics Board (2017) National Ethical Guidelines for Health and Health-Related Research. [Online]

<https://ethics.healthresearch.ph/index.php/phoca-downloads/category/4-neg> [Accessed 30 September 2023].

**Saudi Arabia**

National Committee of BioEthics (2022) Implementing Regulations of the Law of Ethics of Research on Living Creatures. [Online]

<https://researchcompliance.kaust.edu.sa/IBEC/guidelines/Implementing%20Regulations%20of%20the%20Law%20of%20Ethics%20of%20Research%20on%20Living%20Creatures_%20Version%203_2022.pdf> [Accessed 30 September 2023].

**Sierra Leone**

Pharmacy Board of Sierra Leone (2018) Guidelines for Good Clinical Practice (GCP) in Sierra Leone. [Online]

<https://clinregs.niaid.nih.gov/sites/default/files/documents/sierra_leone/PBSL-GCP-Guideline-V2.pdf> [Accessed 30 September 2023].

**Singapore**

Bioethics Advisory Committee Singapore (2015) Ethics Guidelines for Human Biomedical Research. [Online]

<https://www.bioethics-singapore.org/publications/reports/bac-ethics-guidelines-2021/> [Accessed 30 September 2023].

**South Africa**

Department of Health (2015) Ethics in Health Research: Principles, processes, and structures. [Online] <https://www.ul.ac.za/research/application/downloads/DoH%202015%20Ethics%20in%20Health%20Research%20Guidelines.pdf> [Accessed 30 September 2023].

**Sudan**

National Ministry of Health (2008) National Guidelines for Ethical Conduct for Research Involving Human Subjects. [Online]

<https://healthresearchwebafrica.org.za/en/sudan/ethics_doc> [Accessed 30 September 2023].

**Sweden**

Swedish Research Council (2017) Good Research Practice. [Online]

<https://www.vr.se/english/analysis/reports/our-reports/2017-08-31-good-research-practice.html> [Accessed 30 September 2023].

**Switzerland**

Swiss Confederation (2011) Federal Act on Research involving Human Beings. [Online] <https://www.fedlex.admin.ch/eli/cc/2013/617/en> [Accessed 30 September 2023]

**Taiwan**

Ministry of Health and Welfare (2019) Human Subjects Research Act. [Online] [Accessed 30 September 2023].

**Tanzania**

National Institute for Medical Research (2023) Guidelines on Ethics for Health Research in Tanzania. [Online] [**https://nimr.or.tz/wp-content/uploads/2023/06/GUIDELINES-FOR-HEALTH-RESEARCH-2023.pdf**](https://nimr.or.tz/wp-content/uploads/2023/06/GUIDELINES-FOR-HEALTH-RESEARCH-2023.pdf) [Accessed 30 September 2023].

**Thailand**

Forum for Ethical Review Committees in Thailand (2007) The Ethical Guidelines for Research on Human Subject in Thailand. [Online]

<http://www.fercit.org/file/Guideline_English_version.pdf> [Accessed 30 September 2023].

**Turkey**

Ministry of Health of Turkey General Directorate of Pharmaceuticals and Pharmacy (2019) Good Clinical Practice [Online]

<https://www.titck.gov.tr/searchGeneral?term=Good+clinical+practice> [Accessed 30 September 2023].

**United Arab Emirates**

Department of Health (2020) DOH Standard on Human Subject Research. [Online]

<https://nyuad.nyu.edu/content/dam/nyuad/research/hrpp/standard-operating-procedures/ad-doh-rec-procedures.PDF> [Accessed 30 September 2023].

**Uganda**

Uganda National Council for Science and Technology (2014) National Guidelines for Research involving Humans as Research Participants [Online] <https://iuea.ac.ug/sitepad-data/uploads/2021/03/Human-Subjects-Protection-Guidelines-July-2014.pdf> [Accessed 30 September 2023].

**United Kingdom**

NHS Health Research Authority (2020) Governance Arrangements for Research Ethics Committees [Online] <https://www.hra.nhs.uk/planning-and-improving-research/policies-standards-legislation/governance-arrangement-research-ethics-committees/> [Accessed 30 September 2023].

**United States**

Department of Health and Human Services (1991) Federal Policy for the Protection of Human Subjects [Online] <https://www.hhs.gov/ohrp/regulations-and-policy/regulations/45-cfr-46/index.html> [Accessed 30 September 2023].

**Zambia**

National Assembly of Zambia (2013) The National Health Research Act [Online] <https://www.parliament.gov.zm/sites/default/files/documents/acts/Health%20%20Research%20%20Act%202013.pdf> [Accessed 30 September 2023].

**Zimbabwe**

Medical Research Council of Zimbabwe (2011) Ethics Guidelines for Health Research Involving Human Participants in Zimbabwe [Online] <https://mrcz.org.zw/wp-content/uploads/2023/01/Ethics-Guidelines-for-Health-Research-Involving-Human-Participants-in-Zimbabwe-2011.pdf> [Accessed 30 September 2023].

**B: Quantitative coding framework**

| **Variable name** | **Description** | **Code** |
| --- | --- | --- |
| Country | Country name | STRING |
| region | Country region | 1: Africa  2: Asia  3: C. America  4: Carribean  5: Europe  6: MENA  7: N. America  8: Oceania  9: South America |
| Issuer | Document issuer | STRING |
| issuer_type_1 | Organisation type of first document issuer | 1: Government department  2: Research council  3: Regulatory agency  4: University  5: NGO  6: Other (e.g. statutory authority)  7: N/A  99: Missing data |
| issuer_type_2 | Organisation type of second document issuer | 1: Government department  2: Research council  3: Regulatory agency  4: University  5: NGO  6: Other (e.g. statutory authority)  7: N/A  99: Missing data |
| issuer_type_3 | Organisation type of third document issuer | 1: Government department  2: Research council  3: Regulatory agency  4: University  5: NGO  6: Other (e.g. statutory authority)  7: N/A  99: Missing data |
| gov_dept_type | Type of government department issuing the document  Note: Some government department may overlap i.e. Department for Health and Welfare. In these cases, the first category is coded. | 1: Health  2: Education  3: Science/Technology  4: Welfare  5: Other  6: N/A  99: Missing data |
| multiple_issuers | Did multiple organisations issue this document? | 0: No  1: Yes  99: Missing data |
| no_pages | Number of pages in the document | NUMERICAL |
| legal_status | The legal status of the document | 1: Government legislation  2: Guidelines  3: Regulatory requirements  4: Institutional policy/procedures |
| scope | What is the scope of the document?  *Biomedical/clinical trial*: The document clearly only speaks to biomedical researchers and clinical trials  *Implicit:* There is a general obligation for review of human subject research, such that social sciences may be tacitly included  *Explicit:* There is a specific obligation for review in social sciences  *Health-related: There* is an obligation of review in social sciences research that is in some way related to health or biomedical research | 0: Biomedical/clinical trial research  1: Implicit: Social science researchers  2: Explicit: Social science researchers  3: Health-related research  99: Missing |
| vuln_general | Vulnerability discussed (mentioned) | 0: Document does not discuss vulnerability |
|  |  | 1: Document discusses vulnerability |
| vuln_definition | Definition of ‘vulnerability’ | 0: Does not provide a definition of vulnerability. |
|  |  | 1: Does provide a definition of vulnerability. |
| vuln_groups | Specific groups identified/circumstances of vulnerability | 0: Document does not identify specific vulnerable groups or discuss circumstances of vulnerability  1: Document references circumstances in which vulnerability can arise only  2: Document identifies specific vulnerable groups only  3: Both 1 and 2 code conditions are satisfied. |
|  |  |  |
| children | Children identified as a vulnerable group | 0: No |
|  |  | 1: Yes |
| women | Women identified as a vulnerable group | 0: No |
|  |  | 1: Yes |
| preg_fetus | Pregnant women and fetuses identified as a vulnerable group | 0: No |
|  |  | 1: Yes |
| mentally_disabled | Mentally disabled persons identified as a vulnerable group | 0: No |
|  |  | 1: Yes |
| physically_disabled | Physically disabled persons identified as a vulnerable group | 0: No |
|  |  | 1: Yes |
| ethnic_indigenous | Ethnic minorities and indigenous populations identified as a vulnerable group | 0: No |
|  |  | 1: Yes |
| refugees_migrants | Refugees and migrants identified as a vulnerable group | 0: No |
|  |  | 1: Yes |
| prisoners | Prisoners identified as a vulnerable group | 0: No |
|  |  | 1: Yes |
| dependent_relationships | People in dependent relationships identified as a vulnerable group | 0: No |
|  |  | 1: Yes |
| power_hierarchy | People in power hierarchies identified as a vulnerable group | 0: No |
|  |  | 1: Yes |
| elderly | Elderly persons identified as a vulnerable group | 0: No |
|  |  | 1: Yes |
| economic_disad | Economically disadvantaged persons identified as a vulnerable group | 0: No |
|  |  | 1: Yes |
| social_disad | Socially disadvantaged persons identified as a vulnerable group | 0: No |
|  |  | 1: Yes |
| education_disad | Educationally disadvantaged persons identified as a vulnerable group | 0: No |
|  |  | 1: Yes |
| sexual_minority | Sexual minorities identified as a vulnerable group | 0: No |
|  |  | 1: Yes |
| armed_forces | Armed forces identified as a vulnerable group | 0: No |
|  |  | 1: Yes |
| religious | Religious people identified as a vulnerable group | 0: No |
|  |  | 1: Yes |
| conflict | People from conflict zones identified as a vulnerable group | 0: No |
|  |  | 1: Yes |
| sex_worker | Sex workers identified as a vulnerable group | 0: No |
|  |  | 1: Yes |
| cultural_disad | Culturally disadvantaged persons identified as a vulnerable group | 0: No |
|  |  | 1: Yes |
| political_disad | Political disadvantaged persons identified as a vulnerable group | 0: No |
|  |  | 1: Yes |
| rural | People living in rural areas identified as a vulnerable group | 0: No |
|  |  | 1: Yes |
| vuln_researcher | Guidelines discuss need to protect researchers | 0: No |
|  |  | 1: Yes |
| vuln_staff | Guidelines discuss need to protect research staff e.g. interpreters, research assistants etc. | 0: No |
|  |  | 1: Yes |
| dif_consent | Do the national guidelines have a specific separated section for informed consent? | 0: No |
|  |  | 1: Yes |
| dif_writtenconsent | Do the national guidelines specifically require that consent must be provided in a written form? | 0: No |
|  |  | 1: Yes |

**C: Intercoder reliability results**

**C-1: Intercoder reliability – Level of agreement checks**

Cohen’s *Kappa* level of agreement standards.

| **Level of agreement** |  |
| --- | --- |
| Poor agreement | *k* < 0.00 |
| Slight agreement | 0.00 ≤ *k* ≤ 0.20 |
| Fair agreement | 0.21 ≤ *k* ≤ 0.40 |
| Moderate agreement | 0.41 ≤ *k* ≤ 0.60 |
| Substantial agreement | 0.61 ≤ *k* ≤ 0.80 |
| Almost perfect agreement | *k >* 0.80 |

Measurement of agreement results.

| **Variable** | **Cohen’s *Kappa*** |
| --- | --- |
| Vulnerability general | 1.000** |
| Vulnerability definition  Definition type  Definition framing  Vulnerable groups  Children  Women  Pregnant women and foetuses  Mentally disabled  Physically disabled  Ethnic minority/indigenous groups  Prisoners  Power hierarchy groups  Elderly  Economically disadvantaged  Socially disadvantaged  Educationally disadvantaged  Sexual minorities  Armed forces  Religious groups  Conflict-affected groups  Sex workers  Culturally disadvantaged  Politically disadvantaged  Rural  ** p <0.05 | 1.000**  1.000**  1.000**  0.889**  1.000**  0.889**  0.889**  1.000**  1.000**  0.889**  0.889**  1.000**  1.000**  0.889**  0.889**  0.889**  1.000**  1.000**  1.000**  1.000**  1.000**  0.889**  1.000**  1.000** |

**C-2: Intercoder reliability – Coder instructions**

**Task**

**As the secondary coder, you will aid in the measurement of data accuracy by coding a subsection of the overall data set.**

You will view a sample of national research ethics documents and use the coding framework to code several variables. The variables are detailed in the *variables to code* section.

Once you have coded the sample of documents, your finished data will be compared with the data collected by the primary coder to test the coding reliability.

If there are significant inconsistencies in the coding, the primary and secondary coder will discuss the discrepancies and rectify the data if necessary.

**Documents to code**

- You will code nine documents (20% of the total documents). The documents were selected using a random number generator.
- The following country documents were selected:
  - Bangladesh
  - Iceland
  - Malaysia
  - Malta
  - Philippines
  - Singapore
  - Sweden
  - Taiwan
  - Tanzania

**C-3: Stata output – Level of agreement checks**

import excel "/Users/sophiemoxon/Documents/Governing_Research_Ethics/QR_SI/ICR/Cod

> er_1.xlsx", sheet("Sheet1") firstrow clear

(53 vars, 9 obs)

. kap vuln_general C2vuln_general

Expected

Agreement agreement Kappa Std. err. Z Prob>Z

-----------------------------------------------------------------

100.00% 65.43% 1.0000 0.3333 3.00 0.0013

. kap vuln_definition C2vuln_definition

Expected

Agreement agreement Kappa Std. err. Z Prob>Z

-----------------------------------------------------------------

100.00% 50.62% 1.0000 0.3333 3.00 0.0013

. kap definition_type C2definition_type

Expected

Agreement agreement Kappa Std. err. Z Prob>Z

-----------------------------------------------------------------

100.00% 50.62% 1.0000 0.3333 3.00 0.0013

. kap definition_framing C2definition_framing

Expected

Agreement agreement Kappa Std. err. Z Prob>Z

-----------------------------------------------------------------

100.00% 38.27% 1.0000 0.2152 4.65 0.0000

. kap vuln_groups C2_vuln_groups

Expected

Agreement agreement Kappa Std. err. Z Prob>Z

-----------------------------------------------------------------

88.89% 44.44% 0.8000 0.2393 3.34 0.0004

. kap children C2children

Expected

Agreement agreement Kappa Std. err. Z Prob>Z

-----------------------------------------------------------------

100.00% 55.56% 1.0000 0.3333 3.00 0.0013

. kap women C2women

Expected

Agreement agreement Kappa Std. err. Z Prob>Z

-----------------------------------------------------------------

88.89% 88.89% 0.0000 0.0000 . .

. kap preg_fetus C2preg_fetus

Expected

Agreement agreement Kappa Std. err. Z Prob>Z

-----------------------------------------------------------------

88.89% 51.85% 0.7692 0.3243 2.37 0.0089

. kap mentally_disabled C2mentally_disabled

Expected

Agreement agreement Kappa Std. err. Z Prob>Z

-----------------------------------------------------------------

100.00% 50.62% 1.0000 0.3333 3.00 0.0013

. kap physically_disabled C2physically_disabled

Expected

Agreement agreement Kappa Std. err. Z Prob>Z

-----------------------------------------------------------------

100.00% 50.62% 1.0000 0.3333 3.00 0.0013

. kap ethnic_indigenous C2ethnic_indigenous

Expected

Agreement agreement Kappa Std. err. Z Prob>Z

-----------------------------------------------------------------

88.89% 59.26% 0.7273 0.3207 2.27 0.0117

. kap refugees_migrants C2refugees_migrants

too few rating categories

r(499);

. kap prisoners C2prisoners

Expected

Agreement agreement Kappa Std. err. Z Prob>Z

-----------------------------------------------------------------

88.89% 49.38% 0.7805 0.3252 2.40 0.0082

. kap power_hierarchy C2power_hierarchy

Expected

Agreement agreement Kappa Std. err. Z Prob>Z

-----------------------------------------------------------------

100.00% 50.62% 1.0000 0.3333 3.00 0.0013

. kap elderly C2elderly

Expected

Agreement agreement Kappa Std. err. Z Prob>Z

-----------------------------------------------------------------

100.00% 65.43% 1.0000 0.3333 3.00 0.0013

. kap economic_disad C2economic_disad

Expected

Agreement agreement Kappa Std. err. Z Prob>Z

-----------------------------------------------------------------

88.89% 59.26% 0.7273 0.3207 2.27 0.0117

. kap social_disad C2social_disad

Expected

Agreement agreement Kappa Std. err. Z Prob>Z

-----------------------------------------------------------------

88.89% 71.60% 0.6087 0.3068 1.98 0.0236

. kap education_disad C2education_disad

Expected

Agreement agreement Kappa Std. err. Z Prob>Z

-----------------------------------------------------------------

88.89% 88.89% 0.0000 0.0000 . .

. kap sexual_minority C2sexual_minority

too few rating categories

r(499);

. kap armed_forces C2armed_forces

Expected

Agreement agreement Kappa Std. err. Z Prob>Z

-----------------------------------------------------------------

100.00% 50.62% 1.0000 0.3333 3.00 0.0013

. kap religious C2religious

too few rating categories

r(499);

. kap conflict C2conflict

too few rating categories

r(499);

. kap sex_worker C2sex_worker

too few rating categories

r(499);

. kap cultural_disad C2cultural_disad

Expected

Agreement agreement Kappa Std. err. Z Prob>Z

-----------------------------------------------------------------

88.89% 88.89% 0.0000 0.0000 . .

. kap political_disad C2political_disad

too few rating categories

r(499);

. kap rural C2rural

Expected

Agreement agreement Kappa Std. err. Z Prob>Z

-----------------------------------------------------------------

100.00% 80.25% 1.0000 0.3333 3.00 0.0013
